# Preclinical development of an automated injection device for intradermal delivery of a cell-based therapy

**DOI:** 10.1007/s13346-017-0418-z

**Published:** 2017-08-15

**Authors:** Giulia Leoni, Alex Lyness, Patrick Ginty, Rindi Schutte, Gopalan Pillai, Gayatri Sharma, Paul Kemp, Natalie Mount, Michaela Sharpe

**Affiliations:** 1 Cell and Gene Therapy Catapult, 12th Floor Tower Wing, Guys Hospital, Great Maze Pond, London, SE1 9RT UK; 2 Centre for Biological Engineering, Wolfson School of Mechanical and Manufacturing Engineering, Loughborough University, Leicestershire, LE11 3TU UK; 3 Intercytex Ltd, 5 Vale Road, Stockport, SK6 3LE UK

**Keywords:** Intradermal delivery, Automated injection device, Epidermolysis bullosa, Cell therapy, Dermal injection

## Abstract

Current methods for intradermal delivery of therapeutic products in clinical use include manual injection via the Mantoux technique and the use of injection devices, primarily developed for the delivery of vaccines and small molecules. A novel automated injection device is presented specifically designed for accurate delivery of multiple doses of product through a number of adjustable injection parameters, including injection depth, dose volume and needle insertion speed. The device was originally conceived for the delivery of a cell-based therapy to patients with skin wounds caused by epidermolysis bullosa. A series of preclinical studies was conducted (i) to evaluate the performance of the pre-production model (PreCTCDV01) and optimise the final design, (ii) to confirm that a cell therapy product can be effectively delivered through the injection system and (iii) to test whether the device can be safely and effectively operated by potential end-users. Results from these studies confirmed that the device is able to consistently deliver repeated doses of a liquid to the intradermal layer in an ex vivo skin model. In addition, the device can support delivery of a cell therapy product through a customised microbore tubing without compromising cell viability. Finally, the device was shown to be safe and easy to use as evidenced by usability testing. The clinical device has since been granted European market access and plans for clinical use are currently underway. The device is expected to find use in the emerging area of cell therapies and a broad spectrum of traditional parenteral drug delivery applications.

## Introduction

Intradermal (ID) delivery of therapeutic products has gained increasing attention as an alternative route of administration to the conventional subcutaneous, intramuscular and intravenous routes. Some of the advantages provided by targeting the ID layer include minimal invasiveness with the possibility of reduced pain, more rapid pharmacokinetics and a stronger immune response due to the presence of antigen-presenting cells and a dense vasculature [1]. ID injections have been traditionally performed manually via the Mantoux technique, which consists of inserting a fine and short hypodermic needle into the skin at an angle of 5–15° to target the ID layer [2, 3]. Following injection, typically a small bolus should be noticed on the skin surface indicating successful ID delivery. Though extensively used for the administration of vaccines, this method is considered technically challenging and susceptible to variability as it depends on the experience of the practitioner as well as the biomechanical properties of the skin.

Several devices have been developed in recent years with the intention to make ID injections more reliable and user friendly. Some of these systems, including pen injectors, microinjection devices and needle-free injection systems have been made commercially available while others remain in development [3–7]. Here, a novel and versatile automated injection device (CTCDV01) is presented which is capable of delivering multiple and consistent volumes of a liquid medication at dermal and subcutaneous depths. A unique attribute of this device is the possibility to adjust multiple injection parameters, including dose volume, dose speed, needle insertion depth, needle insertion speed and dwell time post-injection. This adaptable system allows for safe injection parameters to be selected based on the specific target skin area and the therapeutic indication.

The initial design concept was to create a delivery system for ID injections in patients affected by epidermolysis bullosa (EB), a group of debilitating inheritable skin disorders characterised by severe skin fragility and blistering. There is at present no definitive cure for this condition; however, a number of cell- and gene-based therapies are under development and showing some early promising clinical results [8, 9]. Notably, fibroblast-based cell therapy has been proposed as an option to restore the deficiency of structural proteins in EB patients. Fibroblasts are the main cell type of the dermal layer where they supply adhesive components of the skin including procollagen and elastic fibres. An allogeneic product containing human dermal fibroblasts (Vavelta, Intercytex Ltd., Manchester, UK) has recently been evaluated in a clinical trial [9]. A single ID injection of this product was reported to improve skin function and accelerate the wound healing process in EB patients [9]. However, a barrier to the routine clinical application of this therapy is the pain and discomfort that patients have reported while receiving injections [6] which appears related to the mode of injection. In order to treat wide regions of wounded skin, ID injections were performed by inserting a 21 gauge (G) needle almost parallel to the skin surface and releasing the cell suspension slowly while retracting the needle (personal communication of Prof John A. McGrath), a practice that the patients found very painful especially in areas of major erosion (Petrof et al., 2013). Development of an alternative method for ID injection in this patient population was therefore considered critical.

The development phase started with defining the device specification for the development of a pre-production model (PreCTCDV01). The experimental work that has been undertaken to evaluate the performance of the pre-production model and inform the design of the final clinical device is presented. First, the ability of the device to consistently deliver repeated doses of liquid to the ID layer in an ex vivo skin model was evaluated. A pilot study was then conducted to confirm that the passage of a cell suspension through a custom-made microbore tubing did not compromise cell viability. Further, a performance study was carried out showing that the device can perform consistent and accurate doses of a GMP-grade cell therapy without detrimental effects on cells. Finally, a formative usability study was conducted, which showed that the device can be safely and effectively used by representative end-users. Despite being designed for a specific medical indication, this device provides a versatile platform for customised delivery of liquid medications, and can be therefore adapted to a variety of dermatological applications for which customised injections are desirable. The device has since been CE marked under the Medical Device Directives (93/42/EEC as amended by 2007/47/EC) in the EU [10, 11], meeting all the essential legal requirements for use in Europe, and plans for clinical use are currently underway.

## Materials and methods

### Injection device specification

**Fig. 1:**
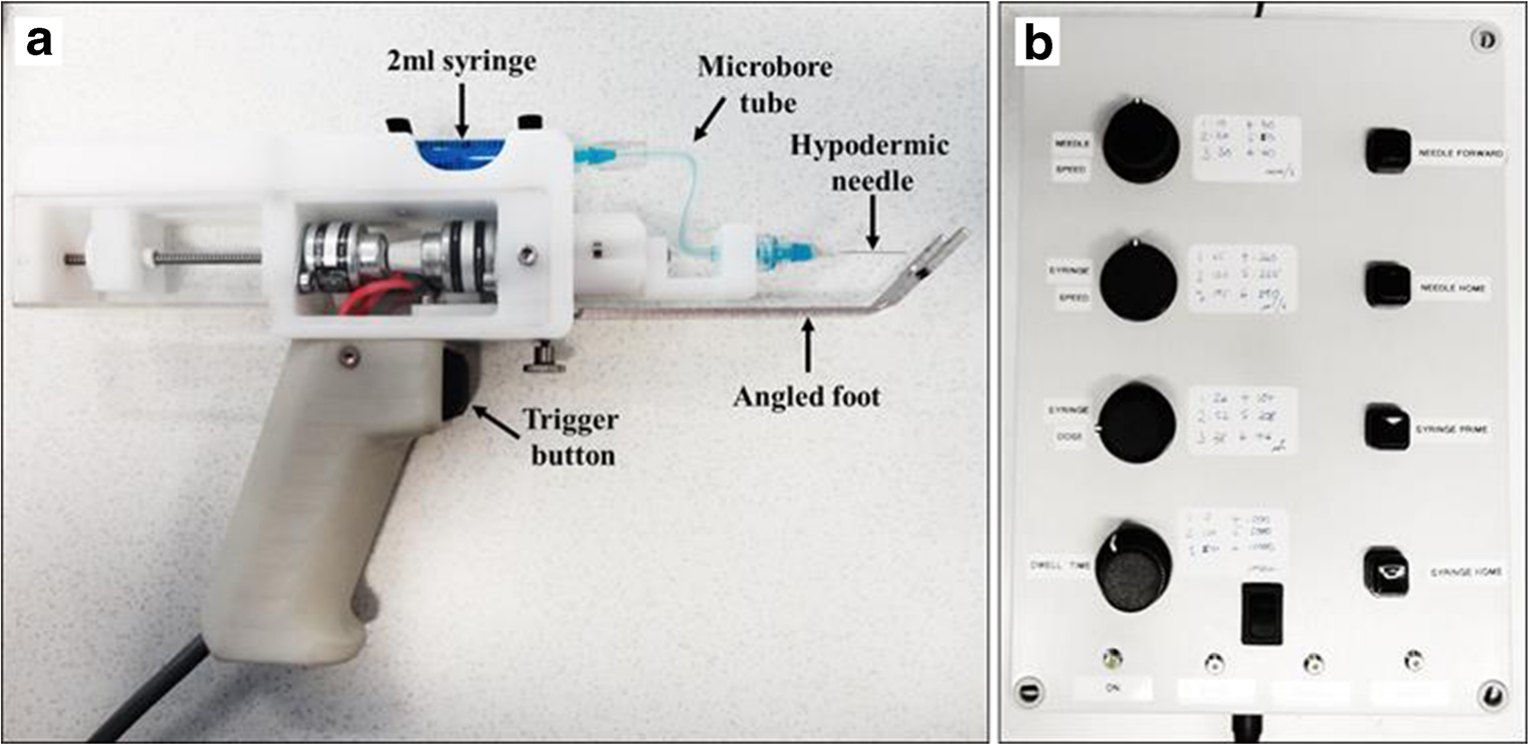
*ProCTCDV01 injection device prototype model*. The device comprises (**a**) a hand-held applicator (**b**) connected to a control box

All studies were performed using a pre-production working device model (PreCTCDV01) manufactured by EG Technology (Cambridge, UK). PreCTCDV01 comprised a hand-held applicator provided with a 30° or 45° angled stainless-steel safety foot and connected through an umbilical cable to a control box (Fig. 1). The function of the safety foot was to allow accurate and steady injections to a fixed depth (see Device operation). The applicator was operated via a control panel on the control box. These included four dials to adjust the following delivery parameters: dose volume (μl) (i.e. the volume of dose to be delivered), dose speed (μl/s) (i.e. the speed at which the dose was delivered), needle insertion speed (mm/s) (i.e. the speed the needle travelled into the skin) and dwell time (s) (i.e. the time that the needle remained under the skin after injection). Four additional buttons were used to prime the system and move the needle forward/backwards (‘syringe prime’, ‘syringe home’, ‘needle forward’ and ‘needle home’). The liquid dose was delivered through a disposable system fixed horizontally on the top of the applicator comprising a 2.5-ml Luer lock syringe connected via a 75-mm long microbore tubing to a standard 27-G, 19-mm (3/4″) hypodermic needle. The microbore tubing (internal diameter 0.5 mm × external diameter 3.1 mm × length 70 mm) was custom-made to fit the device characteristics and has been CE marked to cover its intended use.

### Device operation

The following steps were undertaken prior to each set of injections. The syringe was filled with a solution, either a biological stain (Brilliant Blue FCF, E133) in PBS (1:9 *v*/*v*) to visualise dose dispersal into the skin or a cell suspension containing human dermal fibroblasts, and then connected to the microbore tubing and the needle. The syringe-tubing-needle assembly was manually primed to purge residual air, and the syringe and needle secured into the syringe shuttle and the needle shuttle, respectively. Injection depth, the distance (in millimetres) the needle protrudes from the device, was adjusted manually by aligning the front face of the foot with the tip of the needle and then sliding it back exposing the desired length of needle. All the other delivery parameters were set via the control box. For the ex vivo skin delivery studies, the safety foot was positioned against the delivery surface without applying pressure. The dose was then released by pressing the trigger on the hand-held applicator. Injection was completed once the needle shuttle had retracted.

### Porcine skin

Skin delivery experiments were performed on ex vivo porcine skin, which is an accepted surrogate of human skin due to the similarities in terms of composition, epidermal thickness, permeability and biochemical properties [12–15]. For the pilot study (Study 1: establishment of optimal device configurations for preclinical testing), two skin specimens (~ 20 × 20 cm) were obtained from purchased pork belly cuts, which were frozen at −20 ° and then thawed prior to use. For the preclinical study (Study 2: assessment of the device performance), injections were performed directly on fresh skin of a cadaveric adult pig (large white hybrid, ~ 1 year old), obtained from an unrelated study due to be completed on the same day of the experiment. The Cell and Gene Therapy Catapult has no animal testing facilities in house and all work was outsourced to specialist service providers. The Cell and Gene Therapy Catapult selects appropriate facilities that meet the highest scientific, quality and welfare standards.

### Ex vivo intradermal delivery studies

#### Study 1: establishment of optimal device configurations for preclinical testing

Given the large number of configurations possible with the adjustable settings of the PreCTCDV01, a pilot study (Study 1: establishment of optimal device configurations for preclinical testing) was conducted to identify the most relevant delivery parameters (i.e. foot angle, dose volume, dose speed, injection depth, needle insertion speed and dwell time) and select six candidate configurations for testing in Study 2: assessment of the device performance. A setting was considered relevant when its modification had a significant impact on dose delivery (e.g. optimal vs. non-optimal dose distribution, large vs. small back flow). A total of 24 different configurations (Table 1) were evaluated on the two samples of thawed porcine skin, (sample 1, configurations 1–15; sample 2, configurations 16–24). Each skin sample was pinned to an expanded polystyrene sheet to prevent macro movement. A 10 × 10-cm grid was drawn on each skin sample to trace sites of injections. Five to ten injections were performed for each configuration and qualitative observations recorded after each series of consecutive injections. The device was used free-hand to perform each injection as it is intended to be used in the clinic.

**Table 1:** PreCTCDV01 configurations assessed in the pilot study (see “Study 1: establishment of optimal device configurations for preclinical testing” section)

ID	Foot angle (degrees)	Injection depth (mm)	Needle insertion speed (mm/s)	Dose speed (μl/s)	Dwell time (sec)	Dose volume (μl)
**Sample 1**						
1	30°	2	40	260	1	26
2	30°	3	40	260	1	26
3	30°	4.5	40	260	5	26
4	30°	4.5	40	260	1	26
5	30°	3	40	260	5	26
6	30°	4.5	40	65	5	26
7	30°	4.5	40	390	5	26
8	30°	4.5	40	390	1	26
9	30°	4	40	390	5	26
10	30°	3.5	40	390	5	26
11	30°	4	40	390	5	52
12	30°	4	40	390	2	26
13	30°	4	40	390	4	26
14	30°	4	40	390	3	26
15	45°	4	40	390	4	26
**Sample 2**						
16	45°	4	60	390	4	26
17	45°	3.5	60	390	4	26
18	45°	4	60	390	2	26
19	45°	4	60	390	1	26
20	45°	4	60	390	4	52
21	45°	4	60	130	4	52
22	45°	4	60	130	4	26
23	45°	4	60	130	2	26
24	45°	4.5	60	390	4	26

#### Study 2: assessment of the device performance

The performance of PreCTCDV01 in terms of ability to consistently deliver repeated doses of solution to ID skin depths was further assessed in ex vivo fresh porcine skin (large white hybrid) (Study 2: assessment of the device performance). Seven device configurations identified in the pilot experiment were tested (Table 2). These included a control configuration, which showed optimal dose delivery in thawed pig skin, and six additional configurations obtained by modifying a single parameter at a time: dose volume (μl), dose speed (μl/s), injection depth (mm) and dwell time (s). An angled foot of 45° and a needle insertion speed of 60 mm/s were used for all configurations. To account for skin type variability as expected in a patient population, injections (*n* = 7 per configuration) were distributed throughout the flank area of the animal, where skin characteristics are likely to vary between distinct anatomical sites. After each injection, qualitative assessment of the site of injection was performed and the back flow measured by blotting the solution with pre-weighed blotting paper and weighing it using an electronic balance. Once all injections were completed, skin punch biopsies (~ 2-cm diameter) were collected from each injection site, frozen in pre-chilled isopentane and stored at −80 °C. For histopathology assessment, two consecutive slides were presented per site, one of which was stained with eosin and one unstained. Sections were taken at the point where the injected fluid was at its deepest. Re-sectioning was performed for slides where no visible blue dye could be found on initial examination or where dye was present on the epidermal surface only. A qualitative assessment of the depth of delivery and associated skin region was recorded by a certified pathologist. A manual measurement of the penetration depth was performed and the mean and standard deviations of these data calculated for each configuration.

**Table 2:** Summary of the observation from Study 1 to establish the optimal ProCTCDV01 injection configurations for preclinical testing

Device parameters		Qualitative observations
*Injection depth*	Shallow—2, 3, 3.5 mm Deeper—4 and 4.5 mm	Larger back flow, even at long dwell time Improved delivery and reduced back flow when combined with longer dwell times (4, 5 s)
*Dwell time*	Shorter—< 3 s Longer—> 3 s	Larger back flow Minimal back flow, optimal dose delivery
*Volume*	Large—52 μl	Larger dose delivered but increased back flow
*Dose speed*	From 390 to 130 μl/s	No apparent effect on delivery
*Foot angle*	30°, 45°	No impact on delivery
*Needle insertion speed*	40, 60 mm/s	No apparent effect on injection back flow Higher speed recommended for potential reduction of needle penetration perception

### Cell viability following delivery through a surrogate system

**Fig. 2:**
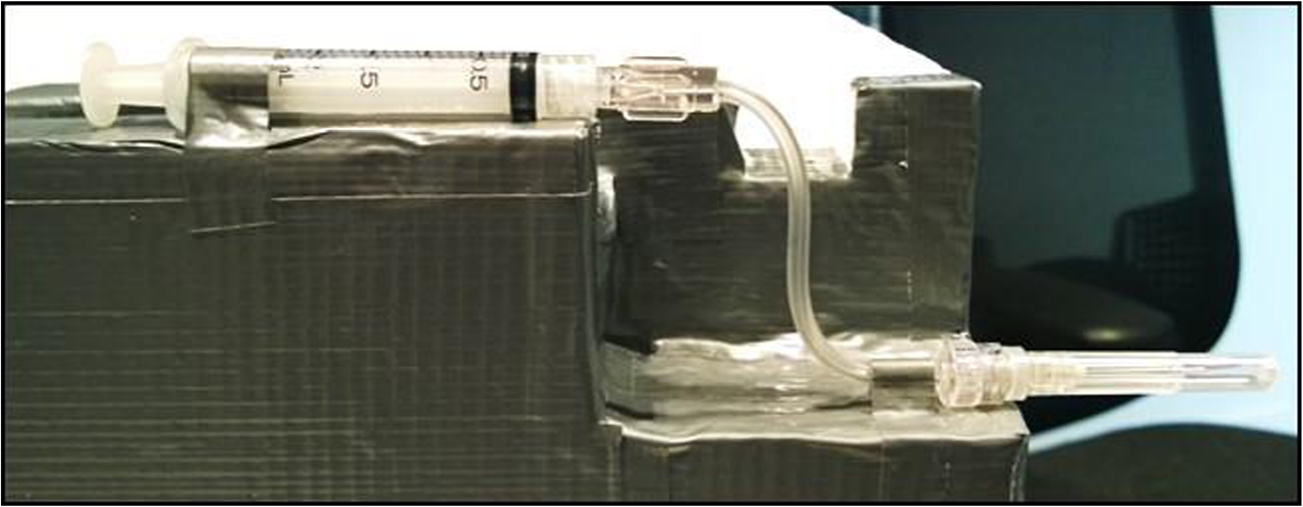
*Surrogate system of the injection device*. The syringe-tubing-needle assembly is mounted on a polystyrene support in the same conformation of the automated device

To evaluate the effect of the microbore tubing on cells, a pilot study was conducted where cell viability was measured following passage through the syringe-tube-needle assembly. For the purpose of this study, a surrogate system of PreCTCDV01 was created by mounting the needle-tubing-syringe assembly on a polystyrene support in the same conformation as the automated device (Fig. 2). A suspension of human dermal fibroblasts (ATCC, Manassas, VA, USA) was prepared to simulate a cell therapy product containing 2 × 10^7^-cells/ml allogeneic human dermal fibroblasts in HypoThermosol-FRS (BioLife Solutions Inc., Bothell, WA, USA). Delivery of cells through a conventional syringe and needle system was used as a positive control. To mimic a multiple dosing scenario, cell viability was measured at different time points following delivery through the surrogate model and conventional syringe and needle. For each experimental group, 2 × 10^7^ fibroblasts/ml (± 10%) were delivered into a cuvette in eight doses of 50 μl, either consecutively or with an 8-min gap between ejections. This time gap between ejections was introduced to account for a potential clinical scenario where a delay between injections may be required during treatment. Cell viability measurements were taken with an automated cell viability analyser Vi-Cell XR (Beckman Coulter, UK) based on the trypan blue dye method.

The effect of temperature and prolonged time within the syringe on cell viability was also evaluated to reflect additional potential scenarios that may occur during patient’s treatment. Three syringes filled with 1 ml of 10^6^ fibroblasts/ml (± 10%) were placed either in the fridge at 2 °C (worst-case scenario for cold environment), room temperature (normal scenario) or in the cell incubator at 37 °C (worst-case scenario for warm environment). Cell viability was measured every 15 min up to 45 min, to represent the worst-case scenario for prolonged treatment delivery. As a control, cells were placed in a cryovial at the same temperatures and viability was measured at the same time points. Residual cell suspension within the tubing following injection was visualised using the EVOS® cell imaging system (Life Technologies).

### Device performance testing using a cell-based product

To establish the dosing performance of PreCTCDV01, a set of experiments was carried out using a GMP-grade cell therapy product containing human dermal fibroblasts (1 ml/vial). The following device settings were used: injection speed = 60 mm/s, dwell time = 1 s and dose size = 26 μl. The dose speed was set to 390 μl/s, with the option to be altered during the study.

In a first experiment, the precision of the device in delivering consecutive 26 μl doses of cell product using a 27-G (210-μm bore size) needle was evaluated. Briefly, the device was fixed to a clamp stand pointing down to allow the dose to be dispensed directly into a weighing vessel placed on a mass balance (Denver Instruments APX-100 Precision Balance) (Fig. 11a). The weight of each dose was used to determine the volume (*n* = 20 doses). After each ejection, the value was recorded and the mass balance tared. As a control test, de-ionised water was used. The cumulative volumes from the first five doses (referred to as ‘first ejections, test 1 (0′)’) was collected and used for cell count and viability measurements using the NucleoCounter NC-3000® (Chemometec). Mean cell diameter was provided by each automated cell count, which was used in combination with cell viability to assess stability of the cell-based product throughout the delivery process.

After completion of the first 20 consecutive doses, the device was left motionless for 60 min to allow cells to settle within the syringe and tubing. Twenty more ejections were performed (referred to as ‘later ejections, test 1 (60′)’) and measurements taken as described above. To further aid understanding of the effect of the cell settling on the device performance, additional measurements were taken on doses obtained from the dead volume in the needle and the tubing (referred to as ‘final ejections, test 1 (> 60′)’), and for cells from a new vial, and ejected after 1-h delay from syringe loading (referred to as ‘first ejections, test 2 (60′)’). In a second experiment, the effect of the needle bore size on the dose volume was investigated using the same procedure as described above. Briefly, the syringe was filled with the cell product and attached to a 25-G needle (260-μm bore size) for the first 20 injections and then replaced with a 22-G needle (413-μm bore size) for further 20 injections leaving the device primed. As a control, viability of the cells drawn straight from the vial and loaded into the NucleoCounter without passing through a 27-G needle was measured.

### Usability study

The objective of this study was to examine whether representative device users (i.e. medical doctors and nurses) could operate PreCTCDV01 in a safe and efficient manner. After receiving training on how to use the device by an experienced study instructor, participants (*n* = 5) were asked to perform three tasks: (i) setting up the device, performing injections on a skin surrogate and changing the needle, (ii) altering device settings i.e. changes of dose volume, dose speed, injection depth and dwell time and (iii) disassembling and cleaning the device. At the end of each task, study participants were requested to complete a User Evaluation Form comprised of 16 questions and provide a feedback on the device. The responses were tallied with a score from 7 points (strongly agree) to 1 point (strongly disagree) to give a result out of 112 points. These were then converted into a percentage score to provide a quantitative measure of user satisfaction with the device. In addition, two study monitors observed the participants completing their tasks and noted any positive or negative behaviour. All the qualitative findings from this study were captured and fed into the final clinical device design, training programme and instructions for use.

### Statistical analysis

All statistical analysis was performed with GraphPad Prism 6 software. The Kolmogorov-Smirnov test was used to assess normal distribution of the data. An unpaired *t* test (for two-group comparison) or a one-way ANOVA (for multiple comparisons) were used for normally distributed data, otherwise a non-parametric Kruskal-Wallis test with Dunn’s correction (for two-group comparison) or the Mann-Whitney analysis were used. A Pearson correlation analysis was conducted to determine the relationship between delivery depth and back flow at the injection site. The null hypothesis was rejected at *p* < 0.05.

## Results

### Identification of a candidate configuration for optimal skin delivery (Study 1: establishment of optimal device configurations for preclinical testing)

For selection of the optimal device setting parameters to be assessed in the ex vivo performance study, a pilot study was conducted. Twenty-four different configurations (Table 1) were evaluated using thawed pig skin (*n* = 2) as a surrogate for human skin. Each configuration was judged on the basis of qualitative observations after dosing, including the amount of back flow from the site of injection following needle retraction and the pattern of fluid dispersal within the skin after it had been sectioned using a scalpel.

A greater volume of back flow was typically observed with shallow injection depths (2, 3 and 3.5 mm), at both high (5 s) and low (1 s) dwell times. Conversely, deeper injection depths (4 and 4.5 mm) resulted in improved delivery and reduced back flow when combined with longer dwell times (4 and 5 s). As expected, injecting twice the volume (52 μl) corresponded in greater back flow from the injection site, suggesting that a smaller volume may be better delivered to the target skin region while ensuring minimal product waste. A different foot angle (and hence injection angle) was also assessed and changing the injection angle from 30° to 45° did not appear to have a significant impact on delivery efficiency. The 45° angled foot was therefore adopted as this enabled better positioning of the device on the skin surface and from an operator experience was the most comfortable.

**Fig. 3:**
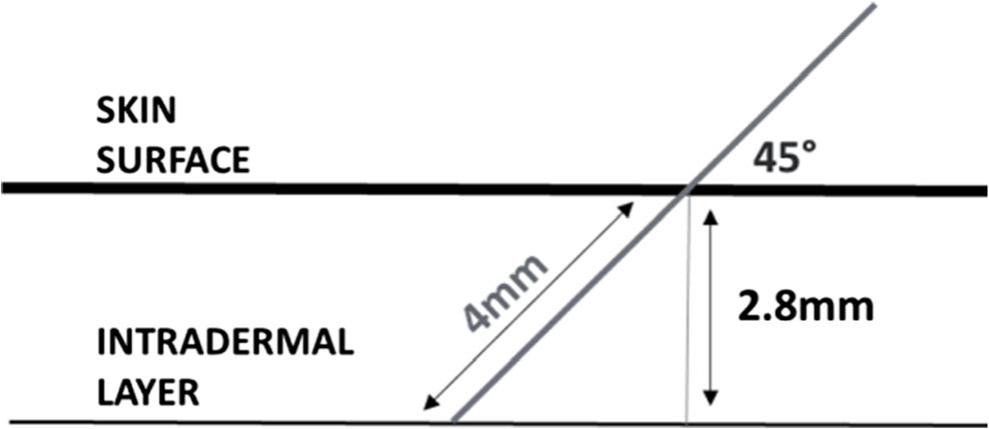
*Schematic representation of the intradermal depth limit calculation*. Using an injection angle of 45° and a delivery depth of 4 mm, the actual needle depth limit when measured perpendicularly from the skin surface is 2.8 mm

Based on these initial qualitative observations, configuration 15 showed consistent delivery of the dye solution into the dermis and minimal back flow. This configuration was modified by increasing needle insertion speed from 40 to 60 mm/s. Back flow from the injected site appeared minimal or unaffected by this change. This parameter was kept at the highest setting (60 mm/s) as a faster needle insertion would ensure the shortest possible injection time in a patient situation potentially reducing their perception of needle penetration. Decreasing the injection depth from 4 to 3.5 mm resulted in an increase of back flow of dye solution from the injected sites. An injection depth of less than 4 mm was hence considered suboptimal for intradermal delivery on this surrogate skin system. With a 45° injection angle and 4-mm injection depth, the actual needle depth limit when measured perpendicularly from the skin surface to the needle tip is 2.8 mm (Fig. 3), which corresponds approximately to the thickness of the human ID layer (~ 2–3 mm) [16]. Based on gross examination of skin cross-section, it appeared that the skin on occasions was not fully breached by the needle penetration, which caused the injectate to leak onto the skin surface.

Variations in needle dwell time showed an effect on dose delivery. Decreasing dwell time to 1 s resulted in a large back flow (15% of dose delivered to the skin based on gross measurements). A dwell time of 2 s did not result in reduction of back-flow volume, indicating that dwell-time values below 3 s may not be sufficient to allow the dose to disperse fully into the target tissue. For configurations with dwell times of 4 and 5 s, minimal back flow was observed with a cut-off point for minimal dwell time between 3 and 4 s. Based on these data, a dwell time of a minimum of 4 s was considered optimal to ensure maximal dose delivery.

Two dose volumes were assessed, 26 and 52 μl. Although injecting twice the volume (52 μl) resulted in a larger dose delivered to the target, the percentage of dose overflow from the injected site was high (50% based on gross measurement), potentially limiting the advantage of higher dose volume delivery at shallow depths. Decreasing the dose delivery speed from 390 to 130 μl/s did not appear to improve delivery of a higher dose, with ~ 50% (gross measurement) of back-flow volume still observed. As a result of the high level of back flow, the lower dose of 26 μl was taken forward as the most suitable setting. In addition, the use of a small dose volume would allow for more injections to be ‘seeded’ across the skin surface, and ultimately for the treatment to be delivered to a more extensive area. A summary of the main observations reported above is presented in Table 2.

**Fig. 4:**
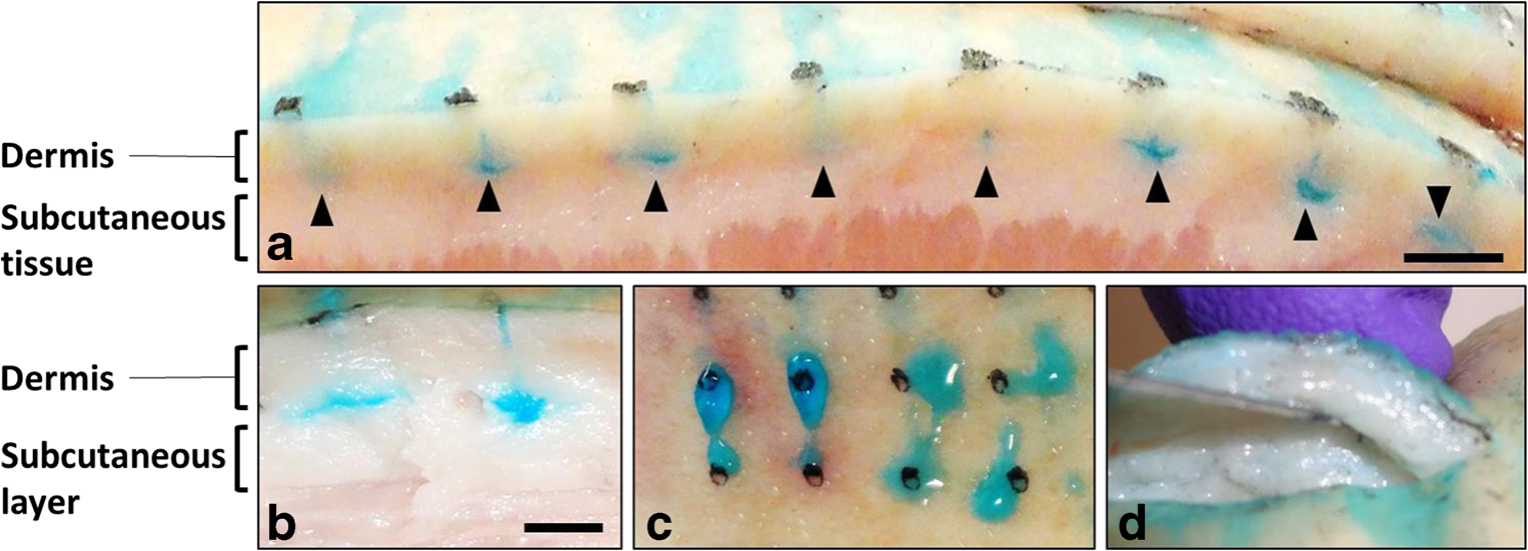
*Representative pictures of injections* via *ProCTCDV01 in* ex vivo *pig skin*. (**a**) Cross-section of pig skin following injection of blue dye using configuration 16, which was selected as optimal configuration for intradermal delivery in this experimental setting. (**b**) Example of delivery to the subcutaneous layer using high injection depth and dwell time (configuration 3). (**c**) and (**d**) Example of back flow at the injection site and lack of blue dye in the expected region of the skin indicating suboptimal dose delivery (configuration 1). *Scale bars* (*a*, *b*) ~ 5 mm)

Representative pictures of efficient and inefficient injections and of the degree of back flow observed are provided in Fig. 4. Altogether, the findings from the pilot study led to the identification of a candidate configuration for preclinical testing which should result in targeted intradermal delivery and minimal back flow from the injection site in ex vivo porcine skin test system. This configuration comprised of the following device settings: 60-mm/s needle insertion speed, 4-mm injection depth, 4-s dwell time, 26-μl dose volume and 45° foot angle.

To provide a comprehensive understanding on how the different injection parameters may affect ID delivery, it was decided that five additional configurations should be evaluated in the preclinical testing (Table 1, configurations 17, 18, 20, 22 and 24; each derived from the control configuration by modifying one of the five device settings at a time (i.e. dose volume, dose speed, injection depth, needle insertion speed and dwell time) (Table 3).

**Table 3:** ProCTCDV01 injection configurations selected for testing in the ex vivo performance study

Configuration ID	Foot angle	Injection depth (mm)	Needle insertion speed (mm/s)	Dose speed (μl/s)	Dwell time (sec)	Dose (μl)	Test
TD1	45	4	60	390	4	26	Control setting
TD2	45	3.5	60	390	4	26	Injection depth (1)
TD3	45	4	60	390	2	26	Dwell time
TD4	45	4	60	390	4	52	Dose
TD5	45	4	60	130	4	26	Dose speed
TD6	45	4.5	60	390	4	26	Injection depth (2)
TD7	45	5	60	390	4	26	Injection depth (3)

Foot angle was kept constant in all the configurations

*TD1* control configuration, *TD2*–*TD7* configurations obtained from the control configuration by changing a single setting at a time

### Preclinical assessment of PreCTCDV01 in a human skin surrogate model

**Fig. 5:**
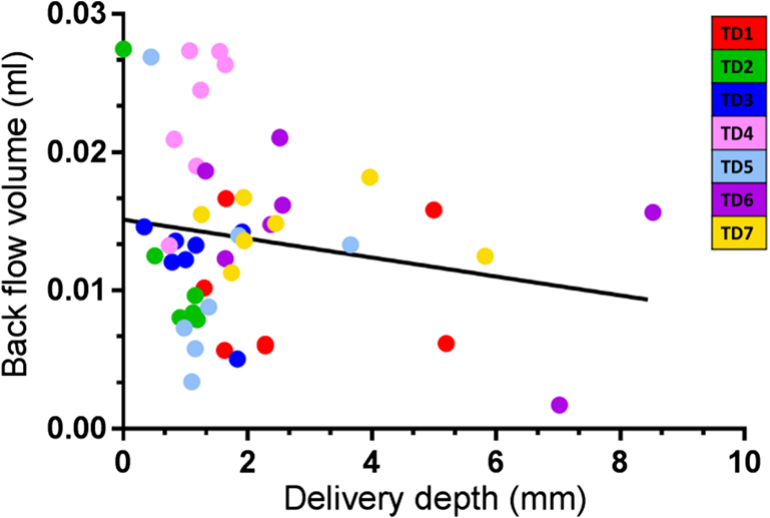
*Correlation between delivery depth and back flow from the injection site*. No significant correlation was observed between delivery depth and back flow (*r* = −0.2)

Fresh excised porcine skin was selected as a surrogate for human skin [17] to evaluate the performance of PreCTCDV01 in terms of ability to deliver consecutive liquid doses intradermally. Quantitative measurements of maximum vertical depth reached by the dye and back flow following each injection were collected and analysed by a certified pathologist. Back flow at the injection sites was variable for each configuration. There was a tendency for the back flow at the injection site to be highest for configuration TD4 (Table 3). This may be expected as the dose volume at this configuration was twice that of the other injections. TD5 resulted in less observed back flow possibly due to the lower dose speed used in this configuration resulting in a slower release and hence increased uptake of the dose into the skin. There was no clear correlation between penetration depth and back-flow volume (*r* = −0.2), although a slight trend towards decreased back flows at higher delivery depths could be observed (Fig. 5).

**Fig. 6:**
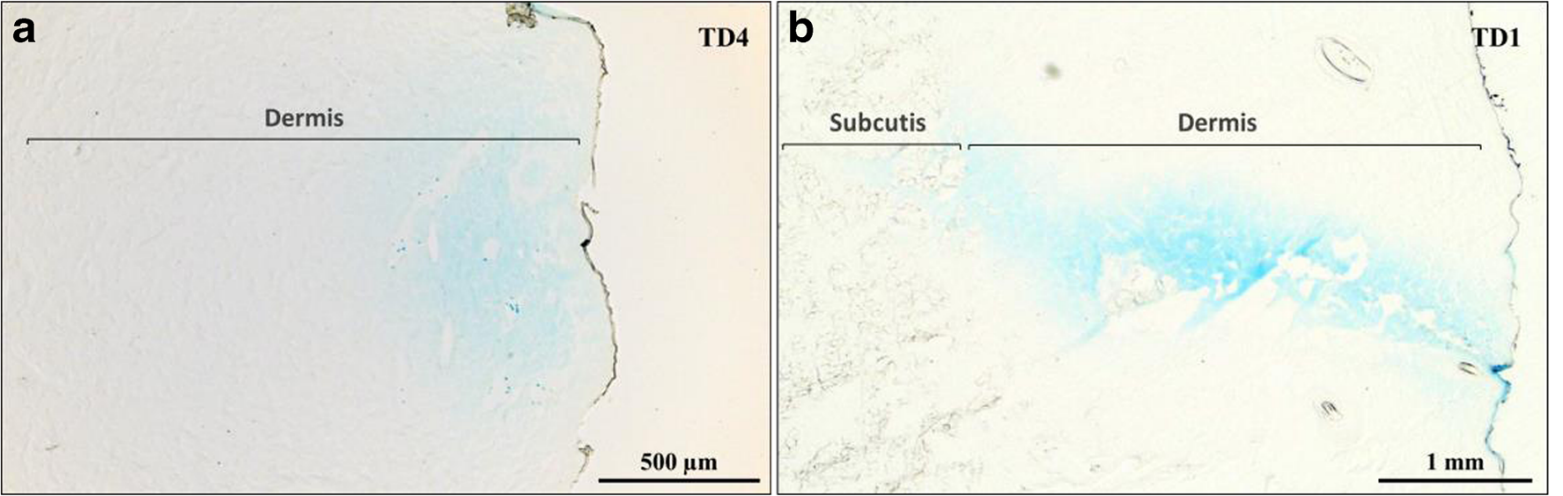
*Histological sections of porcine skin following injection of blue dye with ProCTCDV01*. (**a**) Representative picture of delivery to the intradermal layer and a delivery depth of approx. 500 μm (*black arrow*). (**b**) Representative picture of injection depth in excess of 3.5 mm and achieving subcutaneous delivery (*black arrow*)

**Fig. 7:**
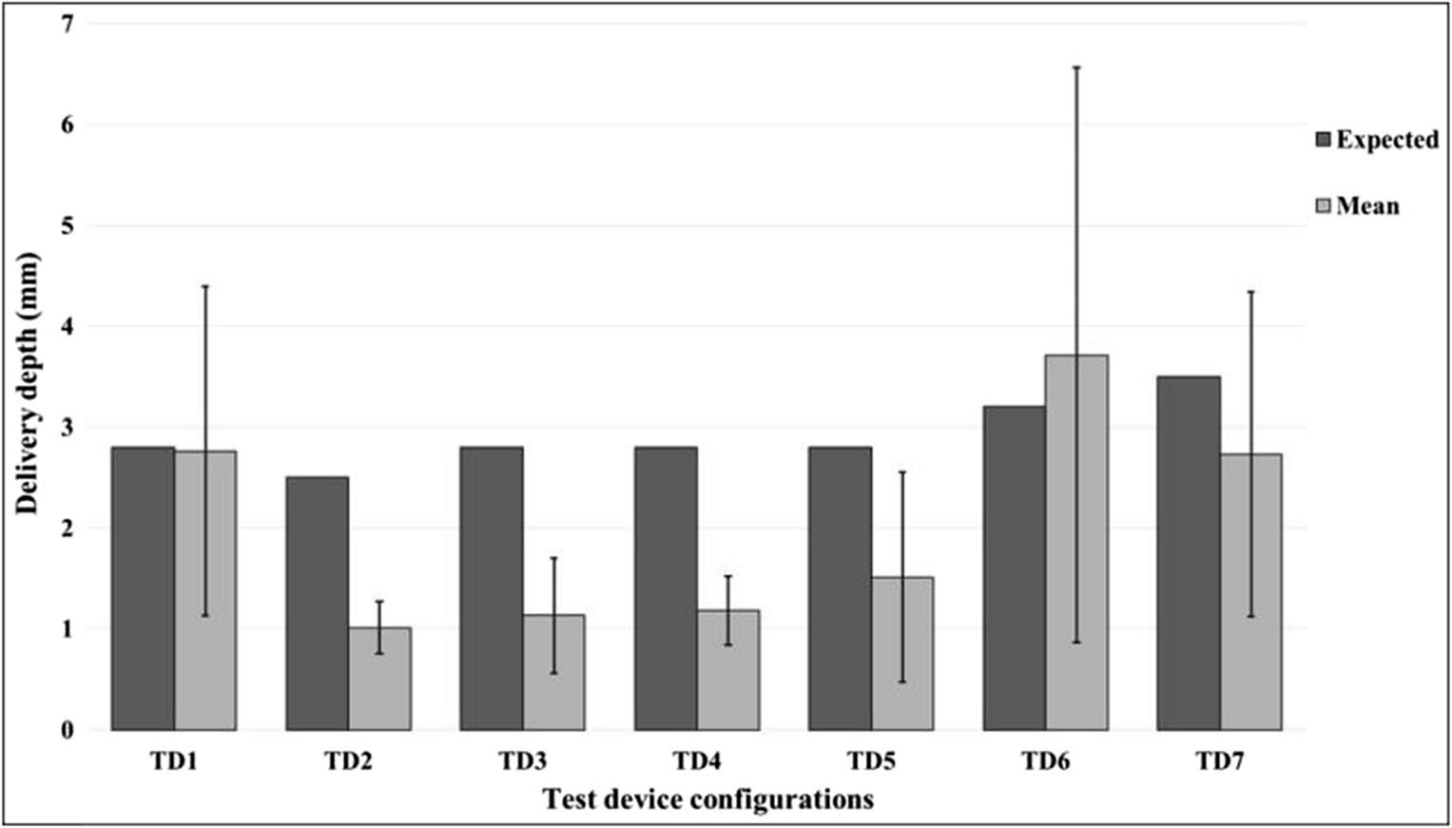
*Comparison of delivery depths between different device configuration*s. A correction factor of + 12.5% has been applied to mean and median values of delivery depth measurements to account for tissue shrinkage (estimated 10–15%) during frozen section histopathology processing [34]

Histological analysis of skin punch biopsies (two consecutive sections per sample) confirmed delivery of the dye solution into the dermis with all device configurations (Fig. 6a, representative image). Dye was also occasionally observed in the subcutis in configurations TD6 and TD7, for which highest injection depths were used, and in configuration TD1 (Fig. 6b, representative image). Overall, the delivery depths achieved did vary, as evidenced by the high standard deviation from the mean for this parameter for many of the device configurations (Fig. 7). This variability is likely a result of the injections being performed throughout the animal’s flank area, where skin characteristics can differ considerably from site to site. Establishing the effect of each configuration on the exact depth of dose delivery was thus not possible.

### Cell viability following delivery though a surrogate system

The effect of the microbore tubing component on fibroblast viability upon injection was assessed using a device surrogate. The effect of the tubing was to be considered detrimental if the mean percentage of viability from test cells (delivered via surrogate system) was statistically lower than that from control cells (delivered via conventional syringe and needle system).

**Fig. 8:**
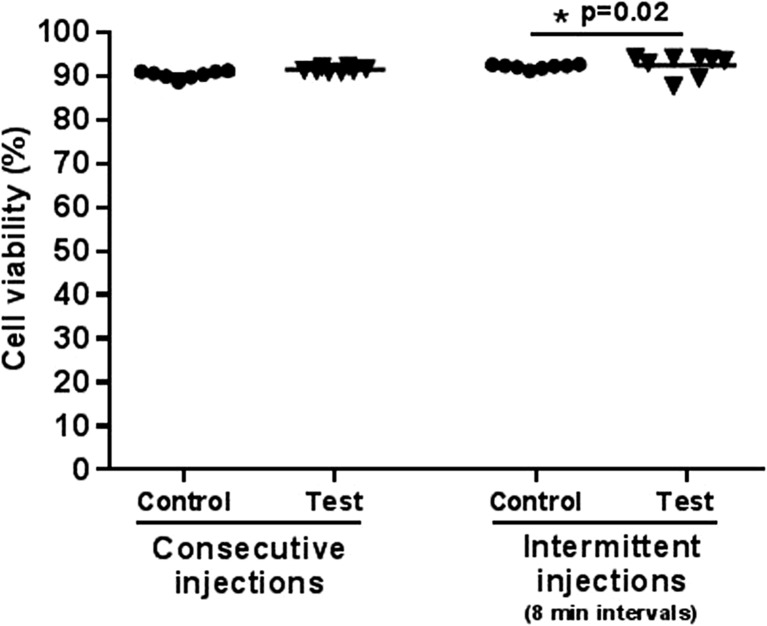
*Effect of the microbore tubing on cell viability*. Viability of a suspension of fibroblasts (2 × 10^7^ cells/ml) was measured upon injection of multiple doses (*n* = 8) through a surrogate system of ProCTCDV01 (test group) and a conventional syringe and needle system (control group). Injections were performed either consecutively or with an 8-min gap. Three independent replicates were performed for each experimental group and data analysed using a *t* test

Overall, average cell viability values from all the experimental groups were above 90%. No significant difference was observed between control and test groups when viability was measured after consecutive injections (Fig. 8, left side of the graph). Similar viability results were obtained when cells were delivered over an extended period of time (Fig. 8, right side of the graph), although the difference between test and control groups was statistically significant (*p* < 0.05). A potential explanation for such difference is the biological variability between different cell batches/flasks, although cells were expanded using the same culture conditions to minimise this factor. As viability was higher in the test group, we can exclude this difference to be due to detrimental effects of the test delivery system.

**Fig. 9:**
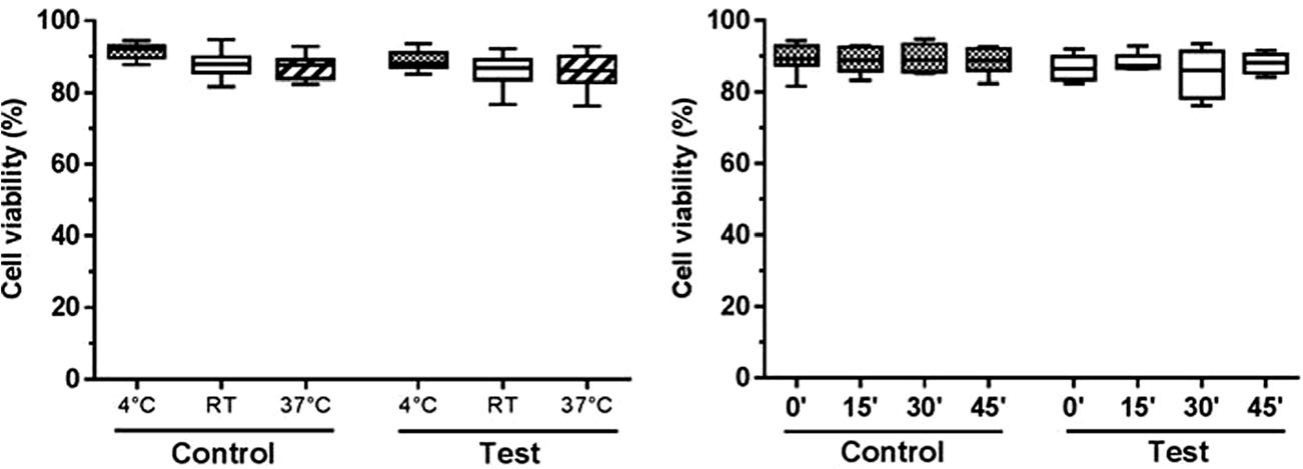
*Effect of temperature and time on fibroblasts viability*. A suspension of human dermal fibroblasts (10^6^ cells/ml) in HypoThermosol®-FRS storage medium was placed in a tube (control) and in a syringe (test) and stored at 4 °C, RT or 37 °C. Viability was measured every 15 min up to 45 min (*n* = 3/temperature condition). No effect of the temperature or time delayed within the syringe on cell viability was observed

The effect of the temperature and holding time within the syringe on fibroblasts viability was also assessed. Test and control cells (cells placed in a syringe or in a tube, respectively) were stored at 4 °C, RT or 37 °C and viability measured at 0, 15, 30 and 45 min. Overall, average cell viability values were above 85%, with no significant differences between and within control and test groups, regardless of the time and temperature conditions (Fig. 9).

**Fig. 10:**
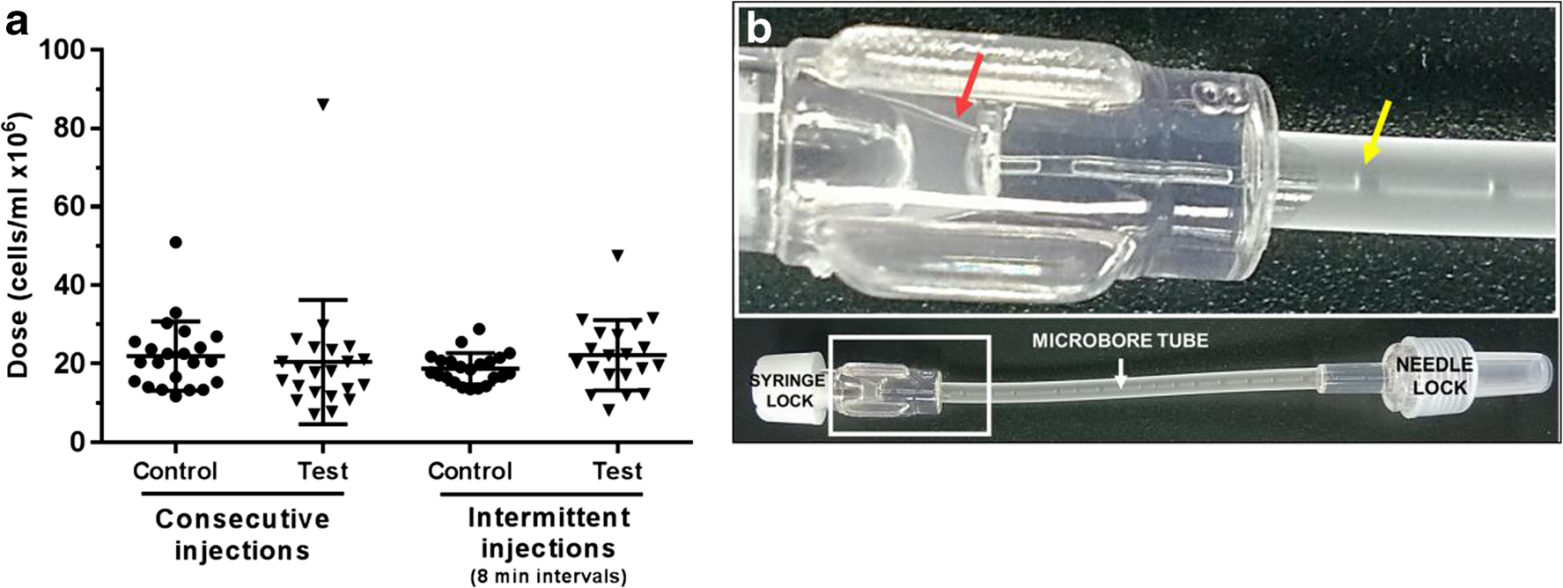
*Effect of the microbore tubing on dose delivery*. (**a**) Cell dose distribution after consecutive and intermittent ejections of a fibroblast suspension (2 × 10^7^ cells/ml) using ProCTCDV01 surrogate system (test) and syringe-needle system (control). Each symbol represents a dose. (**b**) Representative picture of the microbore tube after cell injection. Residual solution and air bubbles can be observed within the syringe adaptor (*red arrow*) and the bore (*yellow arrow*), respectively

Finally, it was investigated whether potential cell clump formation and cell adhesion to the internal surface of the microbore tube could block the system hence affecting dose delivery. Viability results indicated that cells were effectively delivered after each injection with no apparent obstruction of the bore leading to increased cell shearing. Dose concentrations were however variable within each group (SD = 4–16) (Fig. 10a), suggesting that the cell suspension within the syringe was not homogeneous. This was the same for the surrogate device and the control. This may be partially due to the delivery systems being kept still throughout the duration of the experiment, hence allowing time for cells to aggregate and settle within the syringe body. In addition, in the surrogate system, some dead volume was observed within the tube at the end of the dosing, both along the internal bore and within the syringe adapter (Fig. 10a, red and yellow arrows, respectively).

Altogether, these results indicate that the microbore tube can be used to effectively deliver multiple doses of cell therapy product with no apparent detrimental effect on cell viability. It was also confirmed that cells would survive within the syringe for a prolonged period of time and at different temperature conditions, suggesting that a delayed treatment time would be compatible with this type of cell and delivery system.

### Device performance testing using a cell-based product

**Fig. 11:**
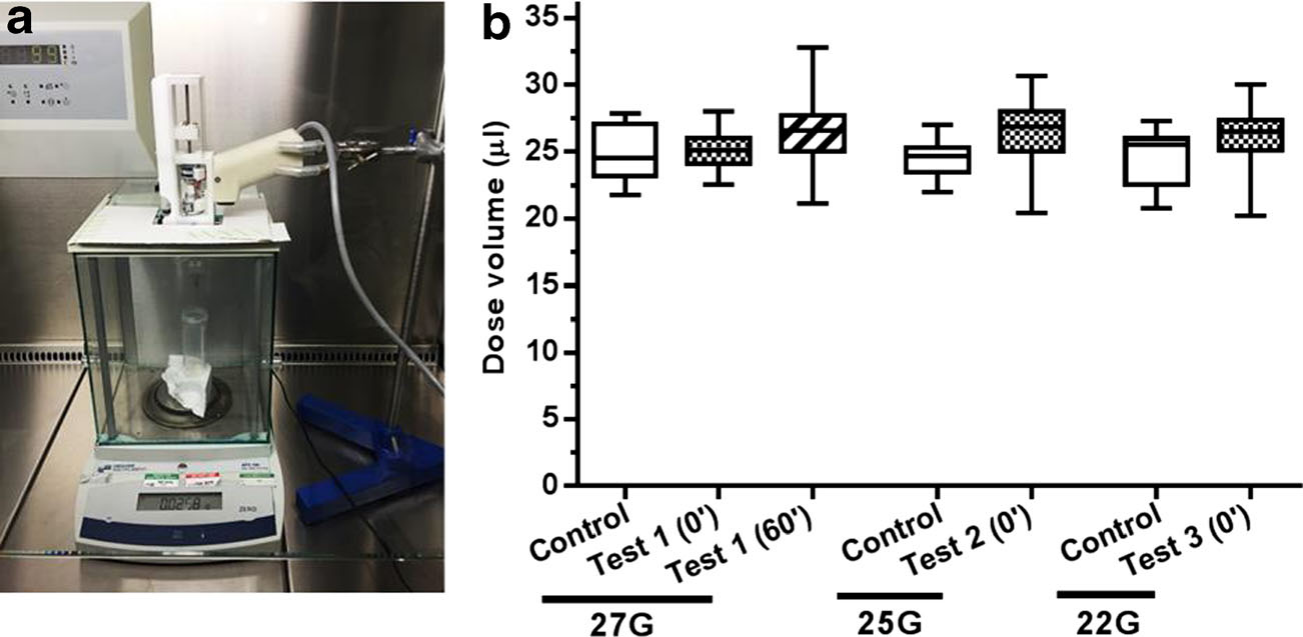
*Device performance testing*. *Effect of time and needle size*. (**a**) Experimental set-up of the equipment used to measure the dose volumes after ejections. (﻿**b**) The graph shows the mean dose volumes of water (control) and cell therapy product (tests 1 and 2) after consecutive (0′) and delayed ejections (60′) and using three different bore size needles

**Fig. 12:**
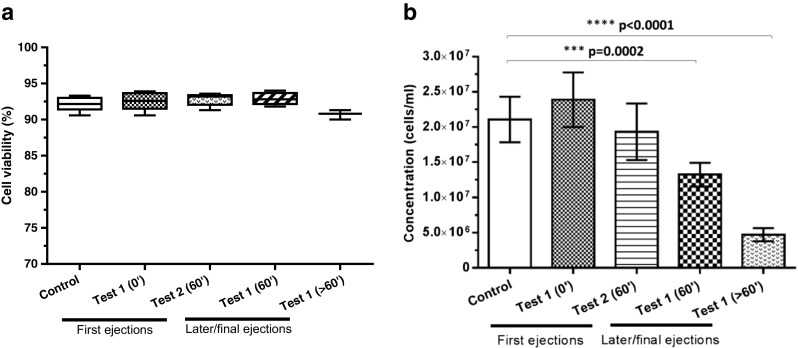
*Device performance testing*. *Effect of time on cell viability and concentration*. Cell viability (**a**) and concentration (**b**) of the cell therapy product were measured following ejection of the cell therapy product using a normal syringe and needle system (control) or the device. Pulled doses from the volume measurement experiment were collected, either immediately after ejections (0′) or after leaving the device stationary for prolonged period of time (≥ 60′), and used for cell parameter measurements

This study was set to measure the device performance in delivering multiple volumes (26 μl) of cell therapy product both immediately and after a 60-min time delay during which the device was left stationary. No significant differences in the mean dose volumes were observed between control and test groups (Fig. 11b, 27-G needle group), with average values ranging from 25.0 μl (± 2.04 SD) for the control group (water), 25.2 μl (± 1.46 SD) for the test 1 group (cell therapy w/o time delay) and 26.5 μl (± 2.72 SD) for the test 2 group (cell therapy with 60-min time delay). Consistent with these results, no significant variations on the dose volumes were observed when delivering the cell therapy product through increased needle bore sizes, with mean values across the three needle gauges (27, 25 and 22 G) of 25.21 μl (± 1.46 SD), 26.38 μl (± 2.91 SD) and 26.15 μl (± 2.46 SD), respectively (Fig. 11b). An assessment of the cells following delivery was also carried out to exclude a potential detrimental effect of the injection system on the cell therapy product. Cell viability was consistently above 90% in all the control and test groups, with no significant difference observed between the control and any of the conditions evaluated (Fig. 12a). Conversely, a decrease in cell concentration was observed in the later and final ejection groups (Fig. 12b). This could be partly a result of the device being left stationary for an extended period of time, allowing the cells to settle towards the side of the needle and resulting in a less homogeneous cell solution within the syringe.

Overall, these data indicate a satisfactory device performance supporting the delivery of a cell-based product, with no evidence of detrimental effects on the cells.

### Usability study results

After assessing the performance of the PreCTCDV01 device in the laboratory, the final study was to perform a usability test to determine how well representative device users could operate the device and gather their feedback with the ultimate goal to identify potential use-related hazards. Participants comprised medical doctors including a dermatologist and trained nursing staff. Results from the questionnaire indicated that participants were overall satisfied with using the device, with an average score of 75%. A more in-depth analysis revealed an average satisfaction score of 62% with respect to device assembly. It was noted that ease of device assembly, and in particular assembly of the needle, tube and syringe components, and manual setting of the injection depth, would be facilitated by providing further support in the form of detailed written instructions and training. In addition, a common concern for all needle and syringe type devices is the risk of needle stick injuries. Following participants’ feedback and discussion, it was concluded that a minor change to the design of the foot would be implemented in the final design of the clinical device in order to minimise the risk of needle stick injuries. This would also improve operator confidence in cases when the device is only used intermittently.

The participants were very satisfied with the injection process and the handling of the device (average score 83%). The disassembly and disposal of the needle and syringe parts were found relatively simple (average score 74%). The participants would also be confident to use the device on a patient’s skin, providing they have received appropriate training prior to use.

Overall, this study led to the following actions which were implemented in the final development stage: (i) preparation of a comprehensive training strategy, including detailed instructions for use with schematic pictures, to ensure correct device assembly; (ii) minor changes to the foot design to improve assembly and further minimise the opportunity for a needle stick injury; (iii) improvement of the injection depth setting method; (iv) a number of improvements to the design of the handset and the control box to facilitate the overall device handling and setting up.

## Discussion and conclusions

A novel automated injection device has been designed that enables consistent accurate delivery of repeated liquid doses to the dermal skin layer and can also target subcutaneous tissue. Unlike manual injection via the Mantoux technique and most of the commercially available systems for ID delivery, this device is provided with multiple injection parameters that can be adjusted according to the intended clinical indication allowing for greater injection control and consistency. The nonclinical development experiments reported here showed that the pre-production device (i) is able to perform injections efficiently and repeatedly into a surrogate model for human skin, (ii) can consistently deliver small doses of a cell therapy product containing human dermal fibroblasts without affecting cell viability and (iii) is safe and has been made easy to use as evidenced by usability testing on representative end-users.

The device specification was initially developed to address an unmet medical need in a patient population affected by a severe skin disorder and for which ID administration of an allogeneic cell therapy product has shown potential therapeutic benefits. As emerged from a clinical trial evaluating this treatment, an issue needing to be addressed was the significant discomfort that patients experienced during manual injection, especially in areas of major erosion [9]. Some of the factors likely contributing to the discomfort in this patient population include the total cell dose volume delivered per injection (i.e. 1-ml fibroblast suspension/injection), the duration and speed of each injection and the length of needle travelling within the skin; some of which cannot be fully controlled when performing a manual injection. While discomfort is highly subjective, the ability to adjust these parameters may ultimately improve patients’ tolerability to the treatment. Importantly, a key feature of this device is that it is minimally invasive and it does not require pressure or a hand to be applied to the skin surface to stabilise the system that can both contribute to further damaging the skin especially when highly aggravated as in EB patients.

The nonclinical studies evaluating device performance were performed on ex vivo porcine skin, which is a widely accepted surrogate model of human skin, as evidenced by a number of dermal penetration studies [14, 18, 19]. Unlike any other laboratory species, pigs have a relatively white, un-pigmented and non-hairy skin and a thick epidermis, which parallel that in man [20]. Notably, the thickness of the porcine flank epidermis (approximately 70 μm) is comparable to the human epidermis [21], and for this reason the flank area was chosen as a target for the ex vivo preclinical study. While in man, the total dermal thickness varies with skin site [22], on the forearm, upper arm and lateral aspect of the thigh it is in the order of approximately 1.3 mm, which is akin to the dermis of the flank of a 3-month-old large white pig (approximately 1.33 mm) [23]. For these reasons, the porcine skin model was considered the most appropriate test system for dermal delivery assessment.

Empirical data collected from the preliminary penetration study led to the selection of a panel of injection parameters expected to affect dermal delivery. These parameters were further evaluated in the pivotal preclinical study on ex vivo porcine skin, where the primary judgement criteria were the consistency of fluid delivery based on histological examination of depth of delivery and back-flow measurements. Data from this study indicated that the device could deliver accurate and repeatable doses to the dermis. Though all configurations performed well, no clear differences could be inferred, likely due to the variability of the penetration depth and back-flow data. This may be partly explained by skin variability between different injection sites, which were randomly distributed on the large flank area of the animal. In addition, while the first set of injections were performed on freshly harvested skin, in the latest injections properties of the skin may have changed. While performing injections in close proximity would have possibly minimised such variability, the study was designed to reflect the variation likely to occur in a patient population. It is in fact well known that skin properties (i.e. dermal/epidermal morphology, biomechanical response, etc.) can vary considerably depending on a multitude of factors [16, 24, 25]. Such differences could be expected or further accentuated in patients with EB or other skin conditions characterised by severely damaged skin.

Another potential source of variability is related to the control scheme used to control the needle and syringe shuttles. However, this hypothesis can be excluded as results from a high-speed camera experiment performed during the initial development phase confirmed that the device can perform consistently at different settings per parameter (unpublished data).

To maximise the therapeutic effect of a cell-based therapy, it is crucial to ensure that cells maintain their functionality and viability throughout the administration process. Multiple factors can contribute to cell damage during the injection procedure and have a negative impact on their viability [26]. Among these, the mechanical forces which cells experience during passage through the needle (i.e. air pressure and shear stress), the needle size and the injection parameters such as ejection rates, have all been suggested to contribute to cell damage during ejection [26–28]. In addition, some cell types may be more susceptible than others to damage and consequent apoptosis, and this in conjunction to the characteristics of the host microenvironment could further contribute to a significant cell loss [29, 30]. For fibroblasts, it has been reported that the percentage of cell damage and consequent reduced viability following positive dispensing via a pen delivery system to be up to 40% [31]. Li et al. investigated the effect of air pressure and other parameters (i.e. needle inner diameter and length) on fibroblast viability by exposing cells to a range of pressures, from normal atmospheric pressure up to a pressure of 500 kPa, which corresponds to the pressure exerted by a syringe pump [32]. No cell damage was observed under these conditions with fibroblasts remaining viable. Here the focus was on the effect of the microbore tubing connecting the syringe and the needle as this custom-made component had not been previously evaluated for the intended use. In addition, the time delay between injections and the temperature at which cells are exposed were considered as additional critical factors for cell dosing in a clinical setting. For the purpose of the study, a suspension of human dermal fibroblasts analogous to the cell therapy product previously tested on EB patients [9] was initially used, where cell concentration (2 × 10^7^ cells/ml), final dose volume (1 ml) and suspension media (HypoThermosol) matched the clinical product [9]. The study findings showed a high and consistent cell viability throughout the dosing procedure, indicating that the microbore tubing is suitable for the delivery of a cell suspension containing fibroblasts. Similar extension tubes made of PVC material are widely used in infusion therapy and in many other different clinical and laboratory applications where high degree of biocompatibility is a prerequisite. Notably, fibroblast viability was neither affected by exposure to relatively low (4 °C) and high (37 °C) temperatures. Similarly, no effect of the time delays between injections on cell viability was detected. From a clinical perspective, this would give the clinician the confidence that the cell product would remain viable within the delivery system even at prolonged treatment times. Another important factor to consider is that cell sedimentation is likely to occur within the syringe and tubing during dosing of multiple injections if the device is left stationary for prolonged periods of time [33]. As a result, the first ejections could contain more cells than those dispensed at the end. This is in line with our study observations. The degree of dose variation between ejections, together with the fact that the system was kept still throughout the experiment, suggested that sedimentation may have occurred within the system. In a clinical setting, this can be easily prevented by inverting the device to ensure the cell suspension is kept homogeneous throughout the dosing procedure. One limitation of this study is that ejections were performed manually using a surrogate system of the device. Although it is possible that the manual ejections were less consistent and may not be truly representative of ejections performed via the automated device, it is important to note the impressive viability results achieved later using the clinical device in combination with a clinical grade cell therapy product [9]. Notably, this additional study confirmed that the device can be employed for the delivery of a cell-based product providing relatively accurate ejections of multiple doses over a prolonged period of time, without detrimental effects on cells as observed in our experimental setting.

Prior to the lock down of the final clinical device configuration, a formative usability test was performed. This study assessed, under simulated conditions, whether the device could be operated safely and effectively by a representative group of end-users and to outline a working practice for safe use of the device. Analysis of the questionnaires and feedbacks indicated an overall positive consensus among participants with regard to device assembly and usability. Prevention of needle stick injuries is paramount for hospital and healthcare workers and an EU directive (2010/32/EU) has come into force providing a framework on how this can be achieved. As a result, a risk assessment for needle handling on the device was considered instrumental to define measures to be integrated into the risk management plan for clinical use of the device. This study also identified the extent of training, both in the form of practical demonstration and written instructions, which should be provided to ensure correct assembly and operation of the clinical CTCDV01 device. Finally, some aspects regarding the design of the device were discussed, including improvement of the foot design to allow easier needle insertion and to automate the set-up of the injection depth.

In conclusion, the preclinical experiments here presented confirmed that the device can perform effectively on an ex vivo skin system enabling targeted release of multiple doses of product by adjusting key injection parameters. Importantly, this device can be operated safely and effectively by potential end-users. This study led to the final stage of the development process, which included building of the clinical devices and regulatory documentation. The device has recently successfully undergone CE mark as a Class IIa medical device through the demonstration of compliance with the EU Medical Device Directive (94/42/EEC as amended by 2007/47/EC) and plans for clinical use are currently under evaluation. While the use of the device in EB patients using a cell-based therapy represents a primary goal, the CTCDV01 device also has the potential to be used across a broad spectrum of traditional parenteral drug delivery applications as well as in other disease areas like critical limb ischemia (CLI) and in scar/wound treatment.

## Abbreviations

ID: intradermal
EB: epidermolysis bullosa
CE: Conformité Européene
